# Natural products are the treasure pool for antimalarial agents

**DOI:** 10.1093/nsr/nwac112

**Published:** 2022-06-14

**Authors:** Bin Zhou, Jian-Min Yue

**Affiliations:** State Key Laboratory of Drug Research, Shanghai Institute of Materia Medica, Chinese Academy of Sciences, China; State Key Laboratory of Drug Research, Shanghai Institute of Materia Medica, Chinese Academy of Sciences, China

## Abstract

Despite the success in malaria control, it remains a life-threatening infectious disease due mainly to the persistent emergence of drug resistance. Sharpened insight into the historical achievements and current trends in antimalarial drug discovery provides more hopes and advantages on natural products for the development of the next antimalarial treatment.

The history of mankind is a constant struggle against diseases, and humans have learned to use natural sources as the remedies to alleviate and/or treat diseases since prehistoric times. The structural diversity and complexity of molecules originated from natural sources are far beyond the scope of human creativity and imagination. Natural molecules with various structures optimized by natural evolution have played an important role in drug discovery, in which the successful antimalarial drug development stands out as a representative example [1–4].

Malaria is a life-threatening infectious disease spread by mosquitoes, claiming millions of lives and causing a heavy economic burden for over millennia. Malaria chemotherapy has a strong historical link to natural products (Fig. 1A), of which quinine and artemisinin are the two most important antimalarial agents from medicinal plants. In 1820, the antimalarial quinine was isolated from the *Cinchona* bark that was traditionally used to treat fever. Although quinine is not currently used as a treatment for malaria because of drug resistance and toxicity, it is still on the World Health Organization's model list of essential medicines for severe malaria. Inspired by the scaffold of quinine, chloroquine was synthesized by the chemists at the Bayer laboratories in 1934, but was shelved due to the obvious toxicity. After a decade of neglect, it was approved as antimalarial for clinical use in 1946 by Food and Drug Administration (FDA) via the US government-sponsored clinical trials. Chloroquine was once considered as the most successful antimalarial drug until the widespread emergence of chloroquine-resistant parasites in the 1950s [4]. In efforts to develop new drugs to overcome the ever-increasing antimalarial drug resistance, artemisinin was discovered in 1972 from a traditional Chinese medicine, the aerial parts of *Artemisia annua* dubbed ‘Qinghao’, which was documented as an antimalarial remedy in 340 CE as per the ancient Chinese pharmacopeia *Principal Prescriptions for Emergency* compiled in China's Eastern Jin Dynasty [2]. Derivatives of artemisinin, including dihydroartemisinin, artemether, artesunate and arteether, were developed in the following decade, constituting the artemisinin-based combination therapies that are the best available treatment for malaria to date [2]. The unique structure of artemisinin makes it distinct from those antimalarial alkaloids in its mode of action, which justifies its high value and efficacy in antimalarial medication. Artemisinin has been found binding to a variety of targets and its exact mechanism of action (MOA) remains to be elucidated. The most accepted theory is that the molecule is activated by haem to generate free radicals, which in turn damage susceptible proteins through alkylation, causing the death of parasites [4].

The historical success of antimalarials from medicinal plants portends that natural products are a treasure pool of new diverse chemotypes and/or pharmacophores for the development of promising antimalarial drugs. In fact, most antimalarials that make up the first-line treatment were derived from or inspired by natural scaffolds, as exemplified by the quinolines, artemisinins and endoperoxides, quinones and quinolones, spiroindolones, etc. [4]. A panel of natural molecules with diverse scaffolds were verified to exhibit distinct activities against the chloroquine-resistant *Plasmodium falciparum* (Dd2, K1), such as febrifugine, sergeolide, yingzhaosu, fosmidomycin, etc. (Fig. 1B), which are important structural templates for the development of novel antimalarial agents [4]. Inspired by the usage of many Chloranthaceae species as traditional Chinese medicine to treat malaria, our research group identified a collection of dimeric lindenane-type sesquiterpenoids (DLS) as nanomolar antimalarial agents for the first time and conducted a structure–activity relationship study [5]. Notably, our breakthrough study led to the discovery of a very potent DLS antimalarial agent sarbracholide, exhibiting an IC_50_ value of 4.3 ± 0.3 pM against the *P. falciparum* strain Dd2 from a Chinese medicinal plant *Sarcandra glabra* subsp. *brachystachys*, which is ∼1000-fold stronger than artemisinin. Particularly, the compound has a high safety index of 9.1 × 10^6^ [6]. Different from all the currently known antimalarial agents, these structurally unique DLS molecules represent a new and promising antimalarial chemotype, which is expected to act through a novel MOA with no cross-resistance to current drugs from the structural point of view.

**Figure 1.**
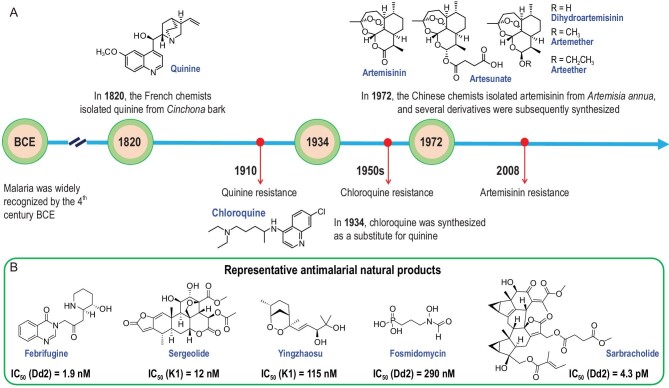
(A) Key events in the historical timeline associated with natural antimalarial drugs. (B) Representative antimalarial natural products.

A lot of studies were conducted to identify drug targets and to understand drug resistance of malaria, resulting in the development of antimalarials with novel MOAs [7]. A quite recent study showed evidence of independent emergence and local spread of clinical artemisinin resistance in Africa [8]. The persistent emergence of drug-resistant parasites to all current medications, especially the global emergence of artemisinin resistant *P. falciparum*, makes it challenging to eliminate and eventually eradicate malaria. Phenotypic high-throughput screening remains an important strategy for new antimalarial chemotypes and many candidates that are currently in development were derived from phenotypic hits, e.g. MMV390048 and KAE609 [9]. Recently, parallel screening approaches including ordinary asexual blood-stage (ABS), ABS apicoplast, liver-stage and/or sexual blood-stage and transmission screenings were developed and applied, which have helped to accelerate the discovery of novel antimalarials [10].

It seems that drug resistance tends to emerge ∼20–40 years after the antimalarial drugs were widely used, such as the cases of chloroquine and artemisinin (Fig. 1A). Therefore, there is a pressing need for discovering new antimalarial agents and/or pharmacophores with novel MOAs, due to the continued emergence of drug resistance to the mainstays of existing therapies. The recent scientific and technological advances in chemistry and biology, especially innovations in the isolation, structural elucidation, chemo- and biosyntheses, drug screenings, as well as chemo- and bioinformatics, facilitate the discovery of new antimalarial agents from natural sources, especially the medicinal plants. Natural products with diverse and unique structures will continue to be the treasure pool for the discovery of new antimalarial treatments.
